# Low acyl gellan gum immobilized *Lactobacillus bulgaricus* T15 produce d-lactic acid from non-detoxified corn stover hydrolysate

**DOI:** 10.1186/s13068-023-02292-5

**Published:** 2023-03-13

**Authors:** Yongxin Guo, Yuru Zhao, Yuan Gao, Gang Wang, Yixin Zhao, Jiejing Zhang, Yanli Li, Xiqing Wang, Juan Liu, Guang Chen

**Affiliations:** 1 College of Life Science, Jilin Agricultural University, Jilin, 130118 China; 2 Key Laboratory of Straw Comprehensive Utilization and Black Soil Conservation, Ministry of Education, Jilin Agricultural University, Jilin, 130118 China; 3 College of Food Science Technology and Chemical Engineering, Hubei University of Arts and Science, Hubei, 430000 China; 4 Sericultural Research Institute of Jilin Province, Jilin, China

**Keywords:** Low acyl gellan gum, Cell-recycle fermentation, d-lactic acid, Cell immobilization, Corn stover

## Abstract

Straw biorefinery offers economical and sustainable production of chemicals. The merits of cell immobilization technology have become the key technology to meet d-lactic acid production from non- detoxified corn stover. In this paper, Low acyl gellan gum (LA-GAGR) was employed first time for *Lactobacillus bulgaricus* T15 immobilization and applied in d-lactic acid (D-LA) production from non-detoxified corn stover hydrolysate. Compared with the conventional calcium alginate (E404), LA-GAGR has a hencky stress of 82.09 kPa and excellent tolerance to 5-hydroxymethylfurfural (5-HMF), ferulic acid (FA), and vanillin. These features make LA-GAGR immobilized T15 work for 50 days via cell-recycle fermentation with D-LA yield of 2.77 ± 0.27 g/L h, while E404 immobilized T15 can only work for 30 days. The production of D-LA from non-detoxified corn stover hydrolysate with LA-GAGR immobilized T15 was also higher than that of free T15 fermentation and E404 immobilized T15 fermentation. In conclusion, LA-GAGR is an excellent cell immobilization material with great potential for industrial application in straw biorefinery industry.

**Graphical Abstract:**

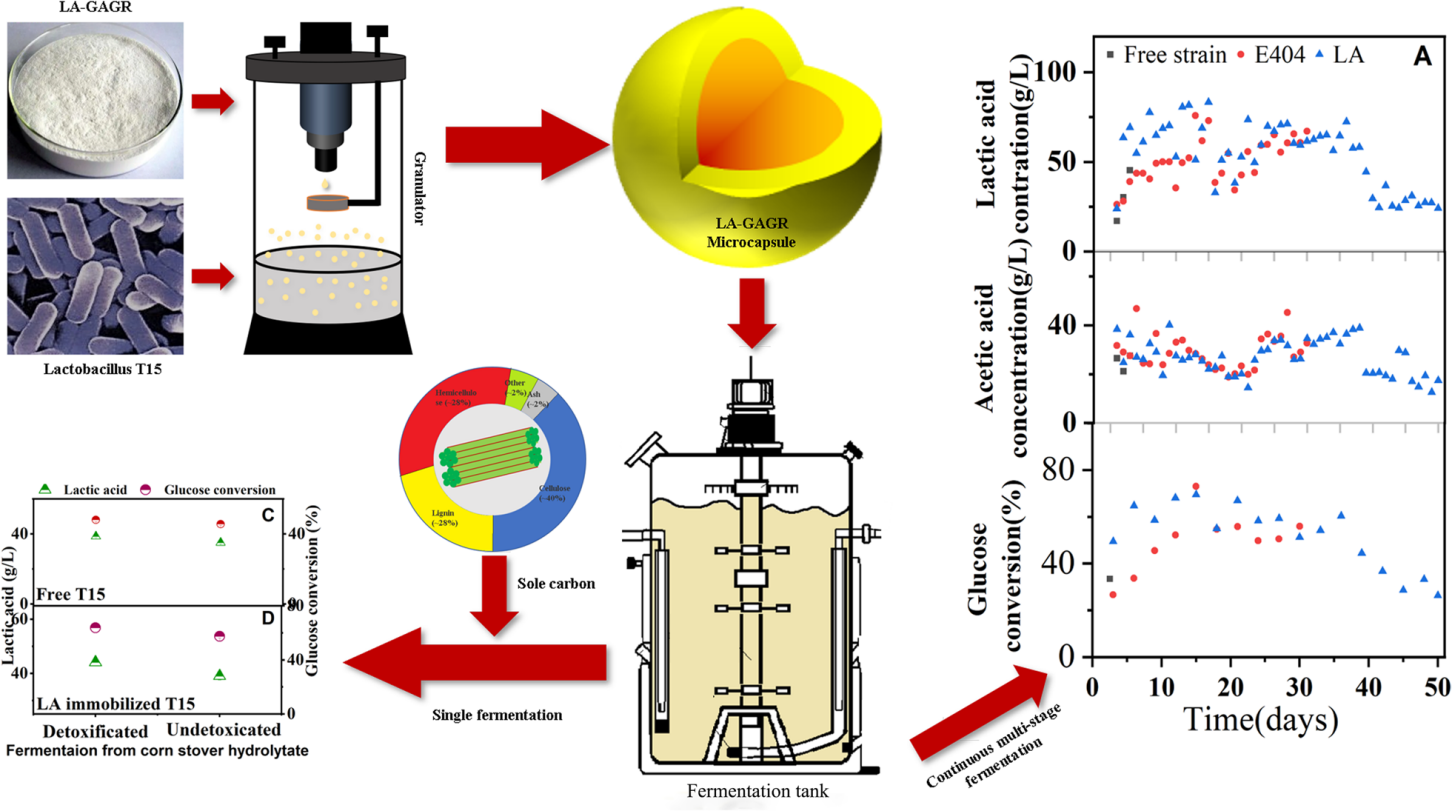

**Supplementary Information:**

The online version contains supplementary material available at 10.1186/s13068-023-02292-5.

## Introduction

d-Lactic acid (D-LA), a chemical isomer of lactic acid (LA), is an important and versatile compound widely used in pharmaceutical, pesticide and chemical industries [1]. It is also the key precursors for the synthesis of stereocomplex PLA which enhances the mechanical properties, thermal stability, and hydrolysis resistance [2–7]. d-lactic acid demand increased sharply with the increase of PLA. Usually, D-LA was produced by microbial fermentation from food or edible resources as substrates, such as corn starch or tomato starch [8]. However, due to the global food crisis getting more and more severe, D-LA production from food resource was obviously undesirable [9–12]. More efforts have been made to find non-human edible materials in biorefinery industry, such as lignocellulosic biomass from agricultural residues [13, 14]. Among them, corn stover (CS) has gained considerable attention as raw material source for producing fermentable sugar which can be used in industrial fermentation field [15]. However, large amounts of phenolic substances (5-HMF, FA, vanillin) that inhibit cell growth were produced in the CS pretreatment process which is the key step of CS to fermentable sugars (Additional file 1: Scheme S1) [16–18]. These inhibitors interact with the cell membrane of the cells, leading to the accumulation of reactive oxygen species and ultimately inhibit the cell growth and metabolism [19]. Although the cells themselves have some self-transformation capacity, they are still unable to undergo complete transformation and are accompanied by a significant loss of ATP during the transformation process [20]. Therefore, how to reduce the toxicity of inhibitors to cells has always been the focus of scientists [21, 22].

Hydrogels are hydrophilic polymers with a cross-linked network structure that can be absorbed and extended under water-insoluble conditions [23, 24]. It was used in various biological and biochemical applications [25, 26]. Low acyl gellan gum (LA-GAGR) is a carboxylic acid functional anionic copolymer composed of D-glucose, D-glucuronic acid, D-glucose and L-rhamnose repeats [27, 28]. Its stability and low ductility of 95% are considered as the main material for biochemical applications [29, 30]. LA-GAGR-based embedded immobilized cell technology is widely used because of its high immobilization rate, high thermal and mechanical stability, ease of extraction, and reusability in continuous reaction cycles [1, 31–35]. Based on the properties of LA-GAGR, we speculated that it could be a very good material for cell immobilization and LA-GAGR could help cells resist the toxic substances (5-HMF, FA, vanillin) in non-toxified corn stover hydrolysate. To verify this hypothesis, we prepared the LA-GAGR immobilized *L. bulgaricus* T15 microcapsules and investigated the utilization of d-lactic acid cell recycle fermentation from corn stover hydrolysates.

## Materials and methods

### Materials

Glucose kit was purchased from Shanghai Rongsheng biopharmaceutical (China), gellan gum with low acyl content, LA-GAGR (Qian Wei Biotechnology), sodium alginate (calcium alginate, E404), Cellic CTec 3 (Novozymes, Enzyme activity: 1000 BHU-2-HS/g), other reagents are analytical grade.

### Strain and cultures

The *Lactobacillus *sp*.* T15 was provided by Key Laboratory of Straw Comprehensive Utilization and Black Soil Conservation, Ministry of Education. The medium used for cultivation was modified MRS medium with pH adjusted to 6.5. The ferment condition for free T15 and immobilized T15 fermentation was static cultivation at 41 °C, 2% inoculum, and 80 g/L initial glucose concentration for 72 h.

### Preparation of LA-GAGR immobilized T15

T15 cultures were added to 300 mL of 1.8% LA-GAGR solution. Encapsulation parameters were kept constant: nozzle diameter of 300 μm; vibration frequency of 120 Hz; syringe pump speed of 3.5 mL/min and the voltage of 1.00 kV. The beads were allowed to gel for 5 min in a gently stirred sterile CaCl_2_ solution, then washed with physiological saline (PS). The immobilized T15 were stored in minimal solution (10% MRS and 90% PS) at 4 ℃ until further use [34]. The preparation of alginate gel immobilized T15 beads used the same process as LA-GAGR immobilization.

### Cell recycle fermentation via immobilized T15 form glucose

LA-GAGR immobilized T15 cell-recycle fermentation was carried out in a 12 mL tube. The fermentation was performed at 37 °C at 80 g/L glucose concentration. After inoculation 6% inoculum of T15/LA-GAGR microcapsules into MRS medium. Take the samples of free cell and immobilized cell recycle fermentation (E404 and LA-GAGR) at 72 h, then determine the d-lactic acid by HPLC. 500 μL of fermentation broth was taken from each cycle, the mass of the remaining gel beads was measured, the concentration of glucose was measured, and the D-LA production efficiency per gram of gel beads was calculated. And the remaining gel column after solid–liquid separation (filtration) was transferred to the same medium as the previous cycle for the next stage of fermentation microcapsules were collected at 0, 9, 18, 27, 36, 45 and 50 days of each cell cycle fermentation and their residual mass was measured to verify the recoverability of the different materials.

### Determination of D-LA

500 μL of 1% H_2_SO_4_ was added in the fermentation broth, shaked and mixed, then centrifuged at 12,000 rpm for 2 min to remove the precipitation. The optical purity of D-LA was determined by HPLC using Astec CLC-L (15 cm × 4.6 nm 5 mm) in a heptane/IPA (9/1) mixture at a flow rate of 1.2 mL/min. The injection volume was 10 µL and the detection wavelength was 254 nm.

### Preparation of detoxified and non-detoxified corn stover hydrolysate

The corn stover pretreatment process was carried out in a 250 mL reactor. Corn stover mass fraction 8%, pretreatment agent composition (NaOH / urea: 8 wt % / 12 wt %), temperature (80 °C) and time (20 min). After pretreatment for solid–liquid separation, the residue was divided into two parts, one of which is rinsed with water to neutral pH (detoxification treatment) and the other part without any treatment. It was then dried at 80 °C for further use. In this study, a solvent system of 2.5 mL citrate buffer (0.05 M, pH 5.0) and 34.8 mL distilled water was used for the enzymatic hydrolysis reaction with 1.579 g of solute from pretreated corn stover. The enzymatic hydrolysis used in this study was based on a designed process: 2.5 mL citric acid buffer (0.05 M, pH5.0) and 34.8 mL distilled water were added to the vessel containing 1.579 g of pretreated corn stover. Adjust pH to 4.6–6.0 and cool down to room temperature, 2.5 mL of cellulase was added, and the samples were preheated in a rotary shaker at 50 °C and stirred at 180 rpm for 72 h. Centrifuge at 3000 rpm for 5 min and collect the supernatant to obtain corn stover enzymatic hydrolysate [36].

### Immobilized cell fermentation form corn stover

LA-GAGR immobilized T15 as the original strain, corn stover enzymatic hydrolysate as the carbon source, fermented at 41 °C for 72 h with static cultivation. D-LA concentration and glucose conversion rate was detected via HPLC.

### Characterization of LA-GAGR with and without T15

#### SEM analysis

High vacuum field-emission scanning electron microscopy was used to characterize LA-GAGR microcapsules. The morphology and surface of the capsules before and after immobilization were observed by SEM (EHT 10.00 kV, Signal = inLens) after the samples were treated with gold plating.

#### Retention analysis

Take riboflavin instead of T15 seed solution, dissolve it in water and stir it at room temperature overnight (keep away from light for the following operations). Prepare 1.2%, 1.4%, 1.6%, 1.8% and 2.0% LA-GAGR, freeze dry, take an appropriate amount of powder and dissolve it in distilled water, stir pan overnight, centrifuge 6000 rpm for 20 min, take the supernatant, measure the absorbance value at 445 nm wavelength with UV–vis spectrophotometer, and calculate the embedding amount according to the formula.

1 $$\mathrm{Embedding\,rate}=\frac{{\mathrm{C}}_{0}}{{\mathrm{C}}_{1}}\times 100\mathrm{\%}$$

C_0_ is the content of riboflavin embedded in gel; C_1_ is the total riboflavin content.

1.2%, 1.4%, 1.6%, 1.8% and 2.0% LA-GAGR were prepared. The gel was added to the 1.5 ml centrifuge tube. After weighing, 12,000 rpm was centrifugated 15 min and the remaining gel was removed. Calculation of water retention of different gels using the lower formula

2 $$\mathrm{Water\,retention\,rate}=\frac{{W}_{2}-{W}_{0}}{{W}_{1}-{W}_{0}}\times 100\mathrm{\%}$$

W_0_: air centrifuge tube quality, g; W_1_: centrifugation before centrifugation with gel tube quality, g; W_2_: centrifugation after centrifugation with gel tube quality.

The TA-XT 2I physical property analyzer is used for uniaxial stress compression test. The fixture parameters were pressure down rate of 1 mm/s, compressive strain 80% until gel breakage. The main parameters used for mechanical strength analysis are fracture strain, fracture stress and Young's modulus. Since the cross-sectional area of the microcapsule will change during the test, it shall be corrected according to formulas (1) and (2) and c model is p/36R and the test mode is compression. The specific test converted into Hencky stress, respectively (σH), and Hencky strain(εH):

3 $$\mathrm{\sigma H}=\frac{{\mathrm{F}}_{(\mathrm{t})}\cdot {\mathrm{H}}_{(\mathrm{t})}}{({\mathrm{H}}_{0}\cdot {\mathrm{A}}_{0})}$$

4 $$\mathrm{\varepsilon H}=-\mathrm{ln}[\frac{{\mathrm{H}}_{(\mathrm{t})}}{{\mathrm{H}}_{0}}]$$

F _(t)_, stress at time t, N; H_(t)_, height of sample at time t, mm; A_0_, initial cross-sectional area of sample, mm^2^; H_0_, initial height of sample, mm.

The fracture stress and fracture strain are σ H- ε Stress and strain corresponding to the highest point of H curve. Young's modulus is σ H- ε which is the slope of the linear part at the beginning of the H curve [12].

The pore size of the gel network can be calculated using Formula (5) [37].

5 $${\upxi } = \left[ {\frac{{3K_b {\text{T}}\left( {{\raise0.7ex\hbox{${{\text{r}}_0^2 }$} \!\mathord{\left/ {\vphantom {{{\text{r}}_0^2 } {{\text{r}}_f^2 }}}\right.\kern-0pt}\!\lower0.7ex\hbox{${{\text{r}}_f^2 }$}}} \right)}}{{\text{E}}}} \right]^\frac{1}{3}$$

ξ Is the aperture, K_b_ is the Boltzmann constant, T is the absolute temperature, E is the young’s modulus obtained in the mechanical property test, and R_0_^2^/R_f_^2^ is the pre factor, can be considered as 1.

### Adsorption of inhibitors (5-HMF, FA, Vanillin) experiment

The original solution was prepared in triplicate from inhibitors (5-HMF, FA, vanillin) and deionized distilled water at a concentration of 2 g/L. The same LA-GAGR (without T15 cells) was added to the solution. The reaction was carried out at 41 °C for 200 min. 50 μL of the solution was taken every 20 min for quantitative analysis using HPLC.

The optical purity of the chiral precursors was determined by HPLC using PRONTOSIL 120–10-C18 H (250 mm × 4.6 mm 10 mm) at a flow rate of 1.0 mL/min. The injection volume was 10 µL and the detection wavelength was set at 285 nm(5-HMF), 321 nm(FA)、280 nm(vanillin).

### Tolerance of LA-GAGR immobilized T15 to 5-HMF, FA, and Vanillin

Microcapsules of immobilized T15 were inoculated into MRS medium containing 5-HMF, ferulic acid (FA), vanillin concentration gradients (0, 0.5, 1, 1.5, 2 g/L) and glucose concentration gradients (60, 70, 80, 90, 100 g/L). The effect of immobilized materials on the tolerance of the strains in adverse environments was studied by changes in their D-LA production.

## Results and discussion

### The reused properties of LA-GAGR immobilized T15 cell in D-LA production from glucose

**Fig. 1:**
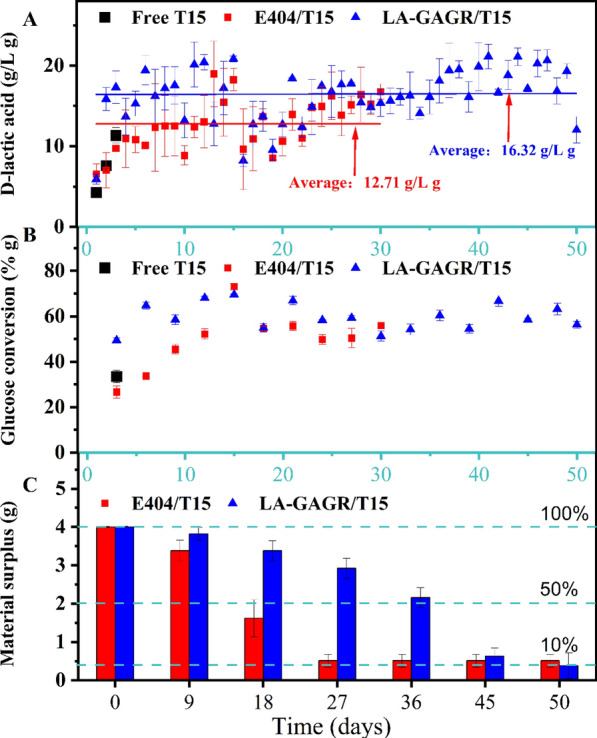
The reused properties of LA-GAGR and E404 immobilized T15 in D-LA production from glucose. D-LA yield of LA-GAGR and E404 immobilized T15 cell recycle fermentation (**A**); glucose conversion rate of LA-GAGR and E404 immobilized T15 cell recycle fermentation (**B**); The weight of the remaining hydrogel of LA-GAGR and E404 immobilized T15 (**C**)

Cell-recycle fermentation experiment was employed to verify the effect of LA-GAGR on D-LA production, glucose conversion in T15 cells, and the longevity of LA-GAGR was verified by the amount of material remaining. LA-GAGR immobilized T15 cells could be used for 50 d, which was 20 d longer than that of alginate immobilized T15 cells (Fig. 1A). We examined the fermentation cycle times of LA-GAGR immobilized T15, which was crucial for evaluating the immobilized materials. LA-GAGR immobilized T15 can be used for 50 days, which is 20 days longer than that of the E404 immobilized T15 (Fig. 1A). The yield of D-LA via LA-GAGR immobilized T15 was 2.77 ± 0.02 g/L h, and the total D-LA production (50 days) was 729 ± 0.28 g/50 d. The average D-LA yield by LA-GAGR immobilized T15 using cell-recycle fermentation was 28.40% higher than that of E404 immobilized T15. The glucose conversion of LA-GAGR/T15 and E404/T15 was all significantly higher than that of free T15 fermentation (Fig. 1B). LA-GAGR immobilized T15 exhibited a longer life cycle with a lower rate of material loss compared to E404 immobilized T15 (Fig. 1C). The average D-LA yield of LA-GAGR immobilezed T15 cell recycle fermentation was 28.40% higher than that of E404. The glucose conversion rate for both LA-GAGR and alginate immobilized T15 fermentation were significantly higher than those of free T15. LA-GAGR immobilized T15 exhibited a longer life cycle and lower loss of material compared to E404 immobilized T15. This proves that LA-GAGR is a perfect immobilized material.

### D-LA production via LA-GAGR immobilized T15 from corn stover hydrolysate

**Fig. 2:**
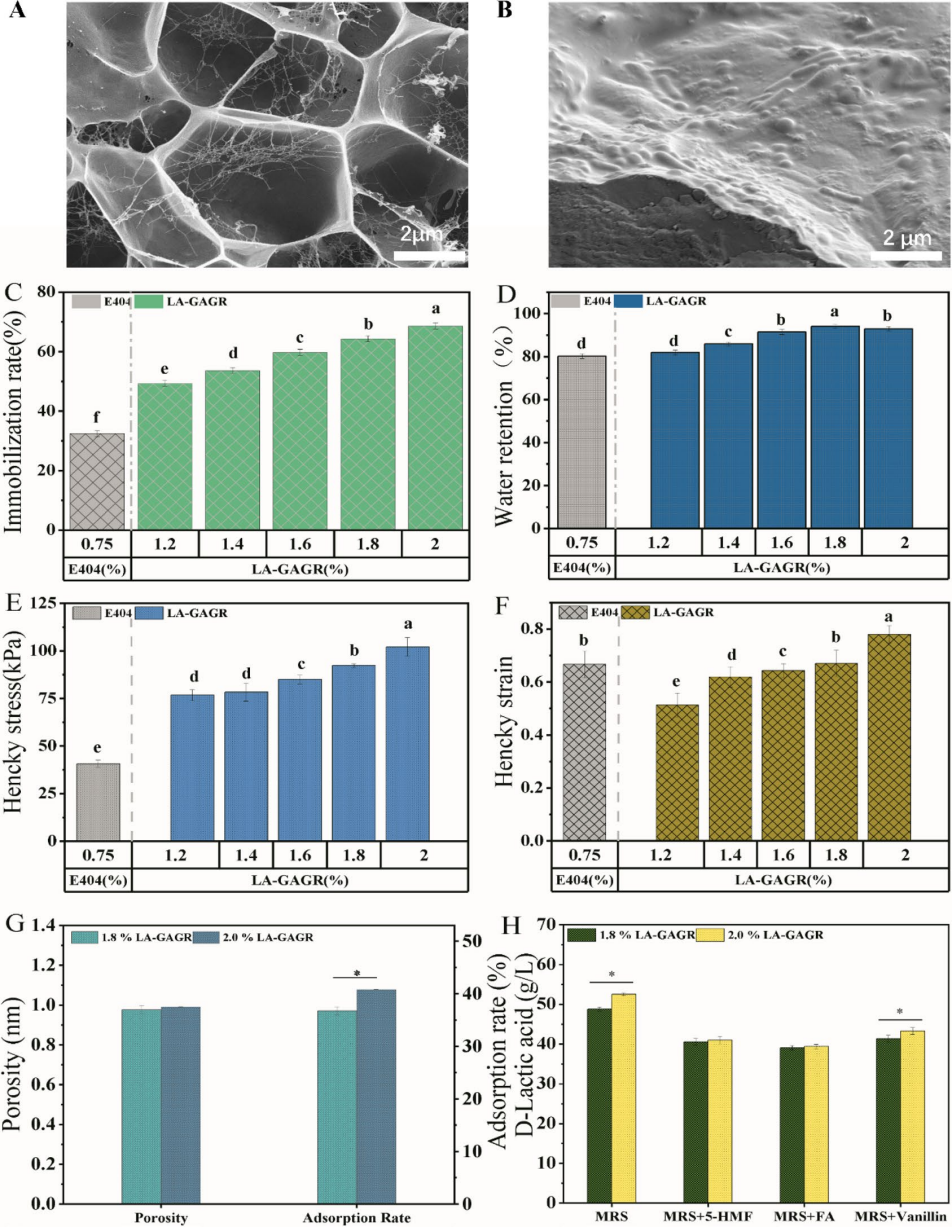
Morphology, mechanical and adsorption characteristics of LA-GAGR and E404. Morphological characteristics of 1.8% LA-GAGR with and without T15 (**A**, **B**), the immobilization rate (**C**), water retention (**D**), Hencky stress (**E**) and Hencky strain (**F**), pore size and adsorption rate (**G**), inhibitors adsorption property (**H**)

To assess the feasibility of cell recycle fermentation of LA-GAGR immobilized cells with corn stover hydrolysate, D-LA fermentation experiment from detoxified and non-detoxified corn stover hydrolysate was employed. The glucose conversion and D-LA yield via free T15 fermentation from non-detoxified corn stover hydrolysate was 48.5 ± 0.9% and 37.95 ± 1.47 g/L, (Table1). However, the glucose conversion rate and D-LA yield by LA-GAGR immobilized T15 fermentation was up to 60.3 ± 2%, and 38.99 ± 0.68 g/L which is higher than that of free T15 fermentation (Fig. 2). The use of LA-GAGR immobilization technology applied to non-detoxified corn stover ferment to produce D-LA is feasible. Also, the D-LA yield of LA-GAGR immobilized T15 was superior to that of E404 immobilized T15 fermentation. The cell recycle fermentation experiment can also be speculated that LA-GAGR has some circularity and continuity in D-LA biorefinery from non-detoxified corn stover hydrolysate.

**Table 1:** D-LA production via immobilized *lactobacillus* T15 from corn stover hydrolysate

	FREE		E404		LA-GAGR	
	Detoxified CS	Non-detoxified CS	Detoxified CS	Non-detoxified CS	Detoxified CS	Non-detoxified CS
Material	Free T15	Free T15	E404/T15	E404/T15	LA-GAGR/T15	LA-GAGR/T15
Titer(D-LA) (g/L)	39.61 ± 1.23^a^	37.95 ± 1.47^a^	43.82 ± 1.24^a^	37.93 ± 1.37^b^	45.07 ± 1.47^**a**^	38.99 ± 0.68^ab^
Yield(D-LA) (g/g)	0.501 ± 0.09^a^	0.472 ± 0.09^a^	0.658 ± 0.01^a^	0.584 ± 0.12^b^	0.736 ± 0.05^a^	0.603 ± 0.02^ab^
Produtivity(D-LA) (g/L h)	0.536 ± 0.05^a^	0.527 ± 0.05^a^	0.608 ± 0.05^a^	0.526 ± 0.05^bb^	0.612 ± 0.04^a^	0.541 ± 0.06^ab^

(1) Means with the different superscript letter in a line differ significantly (*p* < 0.05)

(2) E404/T15, T15 immobilized using E404; LA-GAGR/T15, T15 immobilized using LA-GAGR

(3) Titer (D-LA) (g/L), The amount of D-lactic acid per liter of fermentation liquid at 72 h; Yield (D-LA) (g/g), The amount of D-lactic acid that can be produced per gram of LA-GAGR microspheres at 72 h

### Microscopic characterization of LA-GAGR before and after immobilization of T15

An appropriate LA-GAGR hydrogel pore size is often critical to ensure effective cell immobilization and application stability. The properties of hydrogels are the basis for maintaining their function. Proper concentration of LA-GAGR hydrogel is the key factor to ensure that the cells are immobilized in it completely and the transport of nutrients and metabolites is not affected. When the concentration was 1.8%, LA-GAGR hydrogel exhibited a denser morphology and smaller pores (Fig. 2A, B). The size of *Lactobacillus* T15 is approximately 0.8–1 × 2–20 μm and the molecular size of glucose is 0.55–0.77 nm. The pore size of the microcapsules (0.997 ± 0.04 μm) supports the immobilization of *Lactobacillus* T15 in the gel and allows the glucose and D-LA in and out of the microcapsules (Fig. 2G). The mechanical and water retention properties of the gel are critical for application in the cell-recycle fermentation process. LA-GAGR with different concentrations were investigated for encapsulation rate (%), water retention (%), hencky stress (kPa) and hencky strain. It was found that LA-GAGR of 1.8% and 2.0% exhibited good properties in terms of tensile strength, immobilization rate and water retention properties than E404 and will be a good material for subsequent cell immobilization (Fig. 2C–F). LA-GAGR of 1.8% have the tolerance to 5-HMF, FA, and vanillin and have no significant difference with LA-GARA of 2% (Fig. 2H). The tolerance to the toxic substance of LA-GARA was better than that of E404 (Fig. 2H). So, the LA-GAGR prepared in this study proved to perform better than E404 in the cell-cycle fermentation.

### Adsorption performance of LA-GAGR to inhibitors

**Fig. 3:**
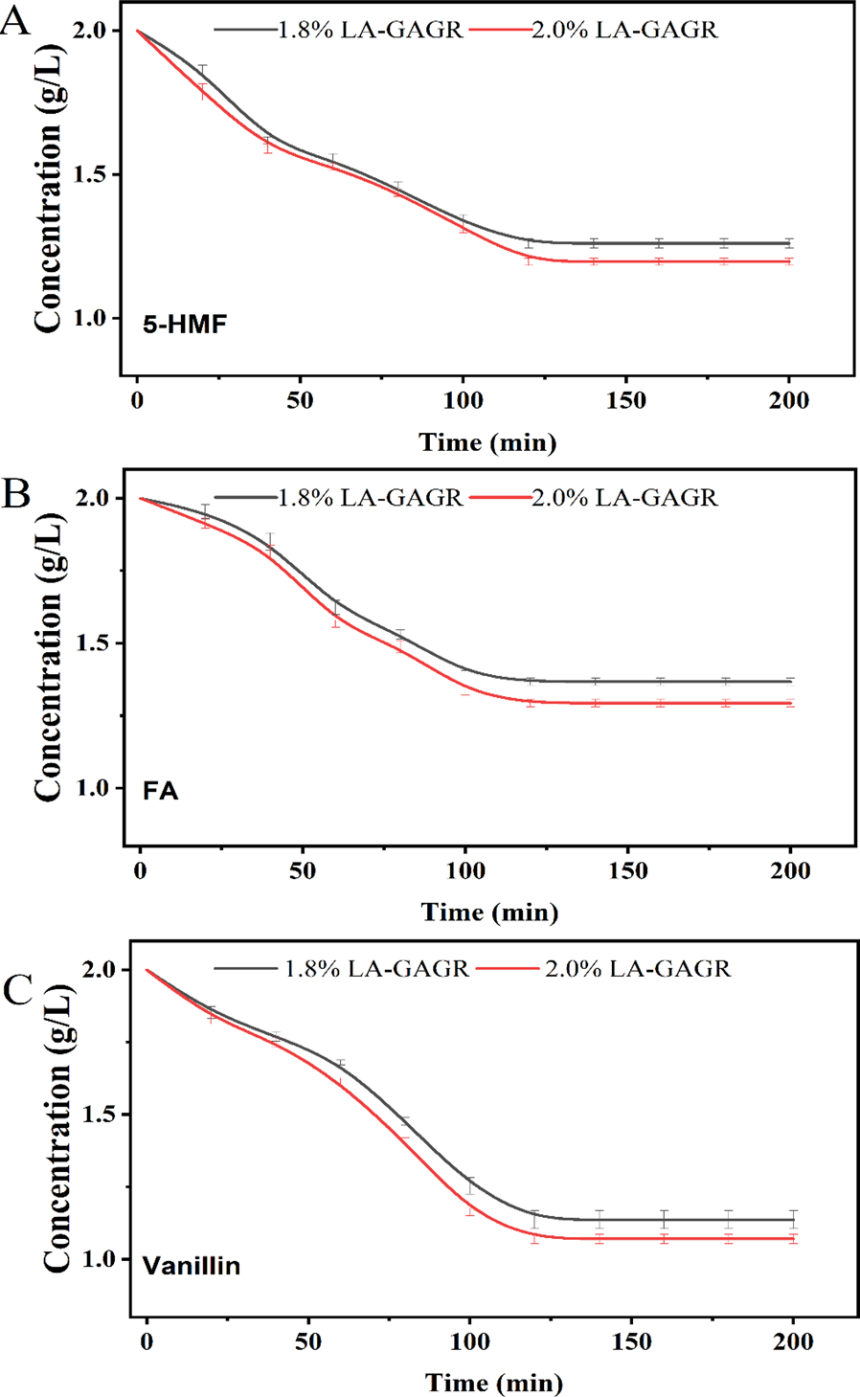
The adsorption capacity of this material was verified by placing different concentrations of LA-GAGR in 2 g/L solutions of 5-HMF (**A**), FA (**B**), and Vanillin (**C**)

LA-GAGR is a kind of porous matrix type material with sub-microscopic lattice structure. This network structure will reduce the permeation rate of cytotoxic substances such as furfural, 5-hydroxymethylfurfural, and polyphenols. It also may own the ability to adsorb small amounts of the toxic substances. These properties protect cells from the toxic substances. It also reduces their accumulation of reactive oxygen species to the cell membrane effectively and improves the yield of D-LA, which was also demonstrated in the study [38]. To study whether LA-GAGR have the ability to adsorb phenolic inhibitors (5-HMF, FA, Vanillin) in the non-detoxified corn stover hydrolysate or not, the adsorption experiment was carried out. LA-GAGR materials at different concentrations (1.8% and 2.0%) was put in a solution containing 2 g/L of the inhibitors, and the results showed that these two materials were able to adsorb the inhibitors and reached the adsorption equilibrium after 2 h with 35–50% adsorption. 2.0% of LA-GAGR was 10% more effective than 1.8% (Fig. 3A–C). This suggested that the concentration of LA-GAGR itself is positively correlated with its adsorption performance at the same temperature and the same initial inhibitor concentration.

### Analysis of tolerance of LA-GAGR to 5-HMF, FA, and vanillin

**Fig. 4:**
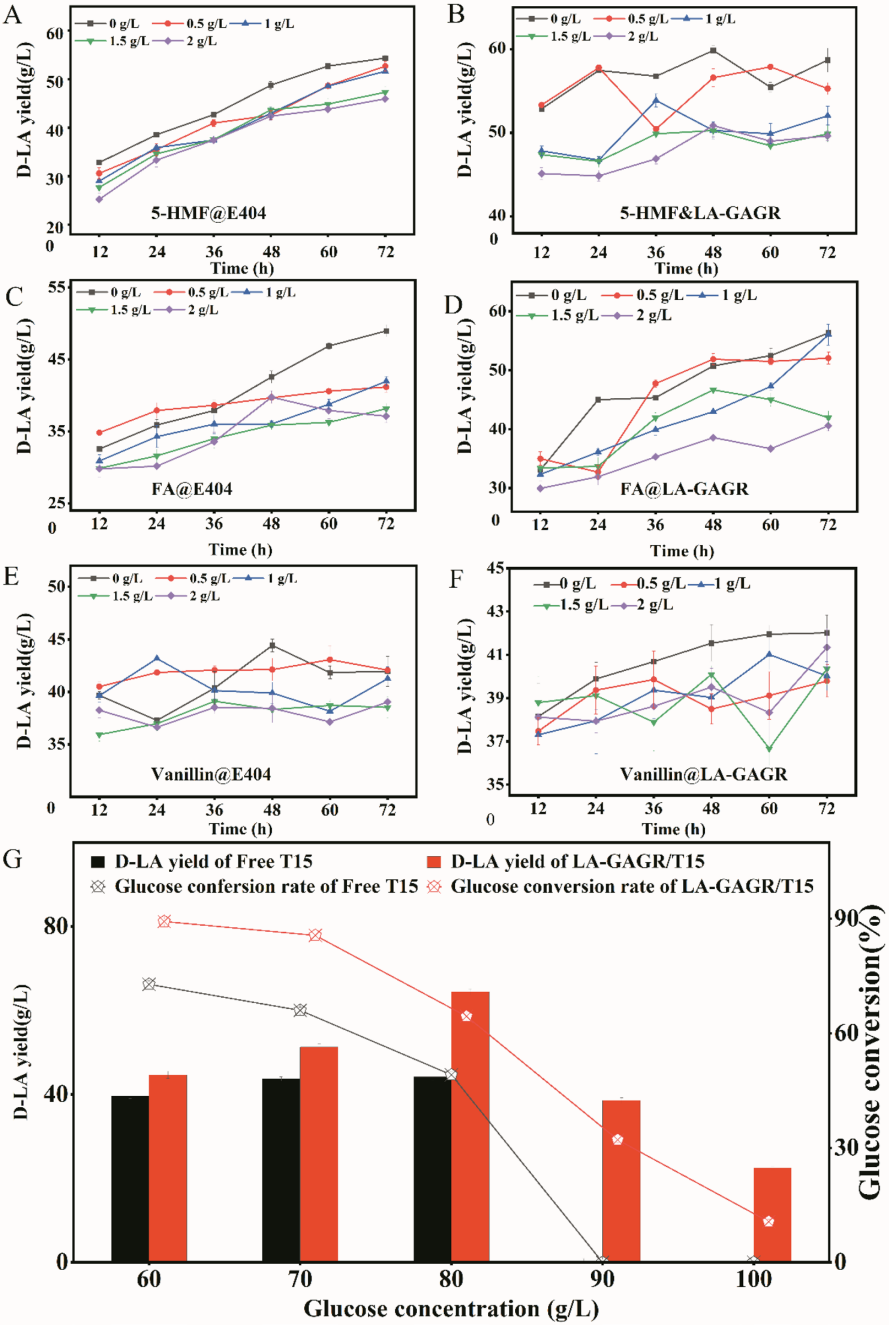
The tolerance analysis of LA-GAGR/T15 (B D F) at 0–2 g/L of 5-HMF (**A**, **B**), FA (**C**, **D**), and vanillin (**E**, **F**) environments, respectively, using D-LA yield as an indicator, and free T15 as a control group for high glucose tolerance analysis (**G**)

Various phenols and aldehydes were produced in the process of corn stover pretreatment which is toxic to cells. Washing with water until neutral or adsorption with activated carbon and resin is the main detoxification method. However, the detoxification process greatly increases the production cost and is one of the main bottlenecks in straw biorefinery. To evaluate whether immobilization of LA-GAGR enhances the tolerance of the strain to the inhibitors during fermentation, 5-HMF, FA, and Vanillin with a concentration of 0–2 g/L were selected for the tolerance experiment. The D-LA production via LA-GAGR immobilized T15 was increased by about 20 g/L compared with E404 in the environment of 0–2 g/L 5-HMF (Fig. 4A, B). T15 with LA-GAGR immobilization showed good fermentation stability at 0–2 g/L FA and vanillin concentration which is higher than that of T15 with E404 immobilization (Fig. 4C, D). When the glucose concentration was more than 80 g/L, LA-GAGR immobilized T15 can growth and D-LA. But free t15 could not grow (Fig. 4G). The results indicated that LA-GAGR prepared in the study as an immobilization material for T15 cells exhibited better tolerance to inhibitors than E404 during D-LA production using undetoxified corn stover hydrolysate. It also means that after LA-GAGR immobilization, T15 improved its tolerance to glucose.

## Conclusion

Hydrogels are gels with water as dispersion medium. In this study, the application field of hydrogel has been broadened. LA-GAGR of 1.8% was superior to calcium alginate E404 in terms of its hencky stress, WHT and encapsulation rate, and was considered as an excellent carrier for immobilization of Lactobacillus lactis T15. After immobilized with LA-GAGR, toxic substances tolerance of T15 increased significantly. LA-GAGR immobilized T15 can be reused for 50 days, which is 20 days longer than that of the conventional E404 immobilized T15. D-LA yield via LA-GAGR immobilized T15 fermentation was increased by 2.6–2.8% compared to E404 immobilized T15 fermentation. This proves that LA-GAGR is an excellent material for cell immobilization again. D-LA yield in this study is at a high level by immobilized cell recycle fermentation [39–43]. This study provides a new idea for Biorefinery industry from corn stover.

## Supplementary Information

**Additional file 1: Scheme 1**. Straw component analysis Straw biomass resources are rich in cellulose, hemicellulose, lignin and other substances. Cellulose can be transformed into glucose through various treatment methods. At the same time, xylose and arabinose synthesized after hemicellulose treatment generate furfural, formic acid, acetic acid and other by-products in microbial cycle, while lignin will produce phenolic substances. The accumulation of the above substances has adverse effects on the growth and breeding of microorganisms). **Scheme 2**. The microcapsule colloid after 4 immobilized T15 using LA-GAGR colloid was inoculated into MRS fermentation medium and cultured at 41 ℃ for 72 h. During this time, the glucose content was assessed every 12 h, and the glucose concentration of fermentation broth was increased. At the same time, the D-LA content was measured by sampling every 24 h. After 72 h, the fermentation broth was changed for the next batch of fermentation, and eventually 17 cycles were continued out.

## Data Availability

All data generated or analyzed during this study are included in this published article.
