# Supplementary material for: Low acyl gellan gum immobilized Lactobacillus bulgaricus T15 produce d-lactic acid from non-detoxified corn stover hydrolysate
Source: Biotechnol Biofuels Bioprod. 2023 Mar 13;16:43. doi: 10.1186/s13068-023-02292-5 (PMC10009946; doi:10.1186/s13068-023-02292-5)
Supplement: Supplementary file 1 — Additional file 1: Scheme 1. Straw component analysis Straw biomass resources are rich in cellulose, hemicellulose, lignin and other substances. Cellulose can be transformed into glucose through various treatment methods. At the same time, xylose and arabinose synthesized after hemicellulose treatment generate furfural, formic acid, acetic acid and other by-products in microbial cycle, while lignin will produce phenolic substances. The accumulation of the above substances has adverse effects on the growth and breeding of microorganisms). Scheme 2. The microcapsule colloid after 4 immobilized T15 using LA-GAGR colloid was inoculated into MRS fermentation medium and cultured at 41 ℃ for 72 h. During this time, the glucose content was assessed every 12 h, and the glucose concentration of fermentation broth was increased. At the same time, the D-LA content was measured by sampling every 24 h. After 72 h, the fermentation broth was changed for the next batch of fermentation, and eventually 17 cycles were continued out. [file 13068_2023_2292_MOESM1_ESM.docx]

**Caption of Scheme**

**Scheme 1**. Straw component analysis Straw biomass resources are rich in cellulose, hemicellulose, lignin and other substances. Cellulose can be transformed into glucose through various treatment methods. At the same time, xylose and arabinose synthesized after hemicellulose treatment generate furfural, formic acid, acetic acid and other by-products in microbial cycle, while lignin will produce phenolic substances, The accumulation of the above substances has adverse effects on the growth and breeding of microorganisms).

**Scheme 2**. The microcapsule colloid after 4 immobilized T15 using LA-GAGR colloid was inoculated into MRS fermentation medium and cultured at 41 ℃ for 72 h. During this time, the sglucose content was assessed every 12 h, and the glucose concentration of fermentation broth was increased. At the same time, the D-LA content was measured by sampling every 24 h. After 72 h, the fermentation broth was changed for the next batch of fermentation, and eventually 17 cycles were continued out.


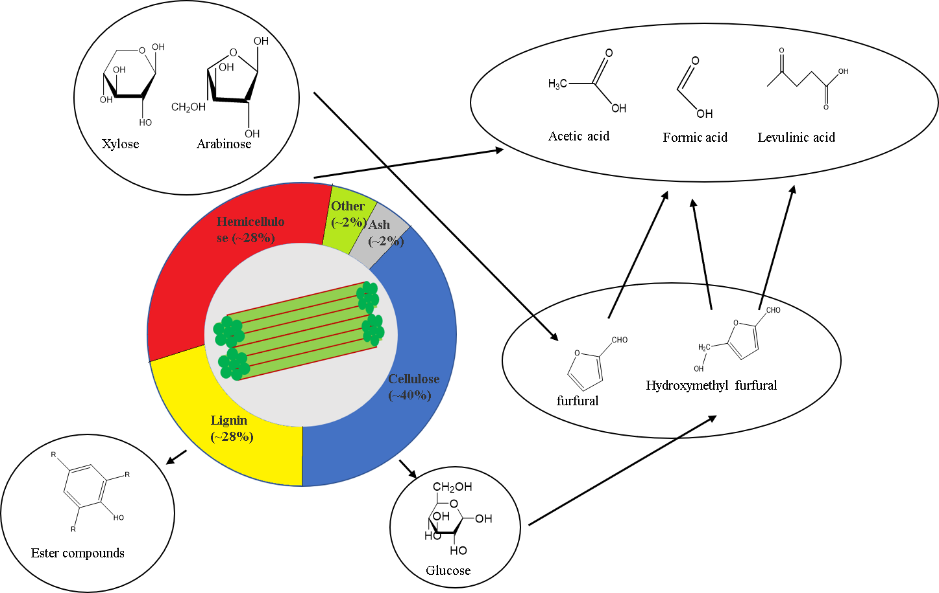


**Scheme 1**. Straw component analysis (Straw biomass resources are rich in cellulose, hemicellulose, lignin and other substances. Cellulose can be transformed into glucose through various treatment methods. At the same time, xylose and arabinose synthesized after hemicellulose treatment generate furfural, formic acid, acetic acid and other by-products in microbial cycle, while lignin will produce phenolic substances, The accumulation of the above substances has adverse effects on the growth and breeding of microorganisms).


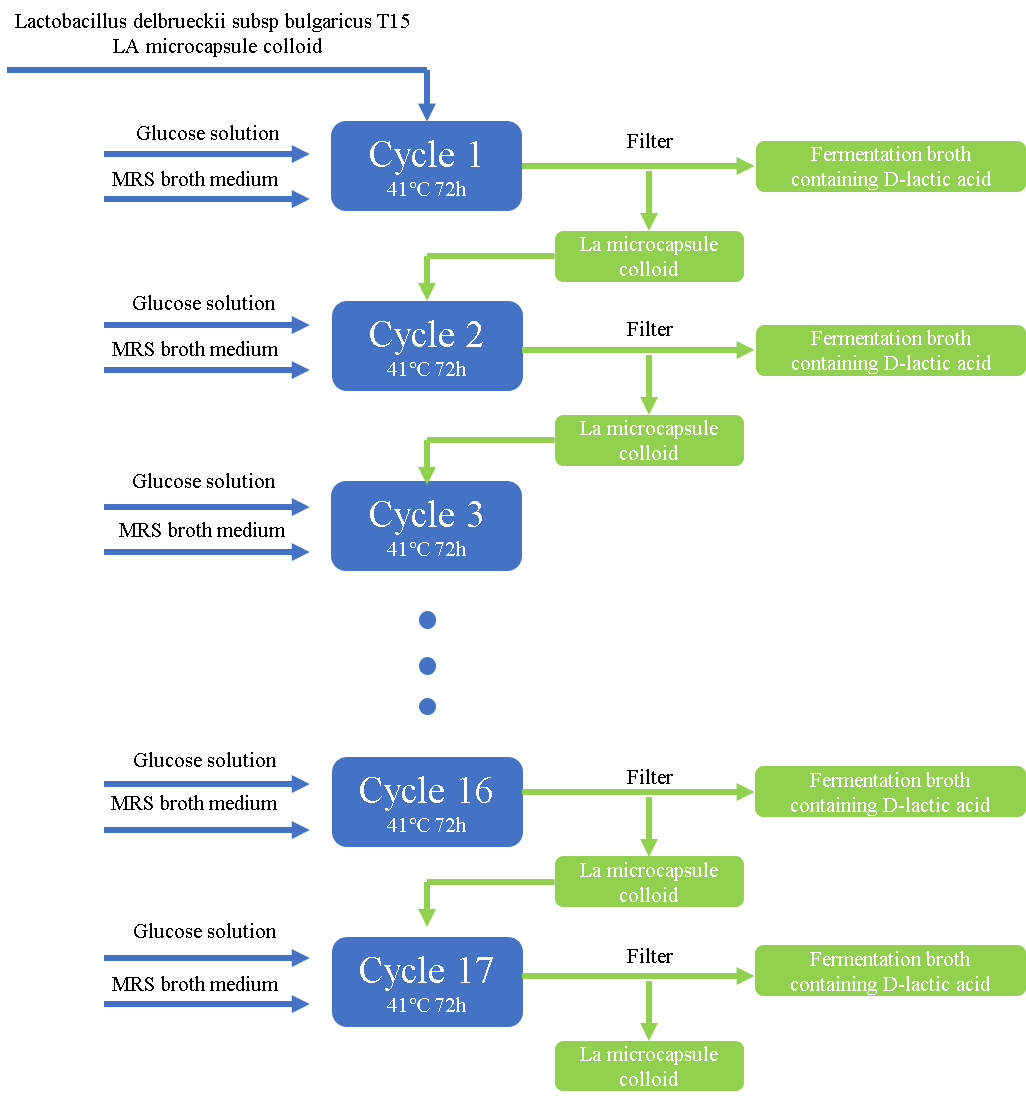


**Scheme 2**. The microcapsule colloid after 4 immobilized T15 using LA-GAGR colloid was inoculated into MRS fermentation medium and cultured at 41 ℃ for 72 h. During this time, the glucose content was assessed every 12 h, and the glucose concentration of fermentation broth was increased. At the same time, the D-LA content was measured by sampling every 24 h. After 72 h, the fermentation broth was changed for the next batch of fermentation, and eventually 17 cycles were continued out
